# Implementing federated learning over VPN-based wireless backhaul networks for healthcare systems

**DOI:** 10.7717/peerj-cs.2422

**Published:** 2024-11-13

**Authors:** Atif Mahmood, Zati Hakim Azizul, Mohammed Zakariah, Samir Brahim Belhaouari, Ayman Altameem, Roziana Ramli, Abdulaziz S. Almazyad, Miss Laiha Mat Kiah, Saaidal Razalli Azzuhri

**Affiliations:** 1 Department of Computer System & Technology, Faculty of Computer Science & Information Technology, Universiti Malaya, Kuala Lumpur, Malaysia; 2 School of Systems and Technology, Department of Software Engineering, University of Management & Technology, Lahore, Pakistan; 3 Department of Artificial Intelligence, Faculty of Computer Science & Information Technology, Universiti Malaya, Kuala Lumpur, Malaysia; 4 Department of Computer Sciences and Engineering, College of Applied Science, King Saud University, Riyadh, Saudi Arabia; 5 Division of Information and Computing Technology, College of Science and Engineering, Hamad Bin Khalifa University, Doha, Qatar; 6 Department of Computer Science and Engineering, College of Applied Studies and Community Services, King Saud University, Riyadh, Saudi Arabia; 7 Department of Computer and Information Sciences, Northumbria University, Newcastle, United Kingdom; 8 Department of Computer Engineering, College of Computer and Information Sciences, King Saud University, Riyadh, Saudi Arabia

**Keywords:** 6G, Wireless backhual, Federated learning, Cross-silo, VPN, Healthcare

## Abstract

Federated learning (FL) is a popular method where edge devices work together to train machine learning models. This study introduces an efficient network for analyzing healthcare records. It uses VPN technology and applies a federated learning approach over a wireless backhaul network. The study compares different wireless backhaul channels, including terahertz (THz), E/V band (mmWave), and microwave, for their effectiveness. We looked closely at a suggested FL network that uses VPN technology over awireless backhaul network. We compared it with the standard method and found that using the FedAvg algorithm with Terahertz (THz) for communication gave the best accuracy. The time it took to reach a conclusion improved a lot, going from 55 seconds to an impressive 38 seconds. This emphasizes how having a faster communication link makes FL networks work much better. Furthermore, a three-step plan was executed to boost security, adopting a multi-layered method to safeguard the FL network and its confidential data. The first step involves integrating a private network into the current telecom infrastructure, establishing an initial layer of security. To enhance security further, licensed frequency channels are introduced, providing an extra layer of protection. The highest level of security is achieved by combining a private network with licensed frequency channels, complemented by an additional layer of security through VPN-based measures. This comprehensive strategy ensures the application of strong security protocols.

## Introduction

In the realm of machine learning, numerous situations present difficulties when it comes to transferring data to a central server for model training. To tackle these issues, an alternative approach is to employ distributed machine learning, as discussed in Verbraeken et al. (2020). Distributed machine learning involves training multiple models on servers located in different geographical locations, and these models are later combined to create a cohesive machine learning model. Two main types of distributed learning strategies exist: the data parallel approach and the model parallel approach. In the data parallel approach, data is divided among various servers, each running the same machine learning model, as explained in Peteiro-Barral & Guijarro-Berdiñas (2013). The model parallel method relies on utilizing identical data across all servers but with distinct parameters for a machine learning model. The distributed machine learning algorithms facilitate quicker learning with shortened training times by employing multiple models on parallel servers, they often neglect the important aspect of safeguarding end-device privacy. The transfer of end-device data to distributed servers can potentially result in privacy infringements on the end-devices. To tackle this issue, the concept of federated learning was introduced in the publication by McMahan et al. (2017).

Federated learning is a concept that has gained significant popularity recently due to its potential in handling fragmented sensitive data for learning purposes. Instead of consolidating data from various sources into a single entity or following the conventional approach of discovering data and then duplicating it, federated learning allows the training of a shared global model using a central server while preserving the data within local clients where it originates (Qayyum et al., 2022).

### Federated learning in healthcare.

Healthcare data often appear fragmented due to the intricate structure of the healthcare system and its associated procedures. For instance, various hospitals may have access exclusively to the clinical records of their respective patient groups. These records contain highly sensitive information, including protected health data (PHI) of individuals. To govern the access and analysis of such data, stringent regulations like the Health Insurance Portability and Accountability Act (HIPAA) have been established (Gostin, 2001). This presents a significant obstacle for contemporary data mining and machine learning (ML) methodologies, including deep learning (LeCun, Bengio & Hinton, 2015), as they typically rely on substantial volumes of training data.

**Figure 1:**
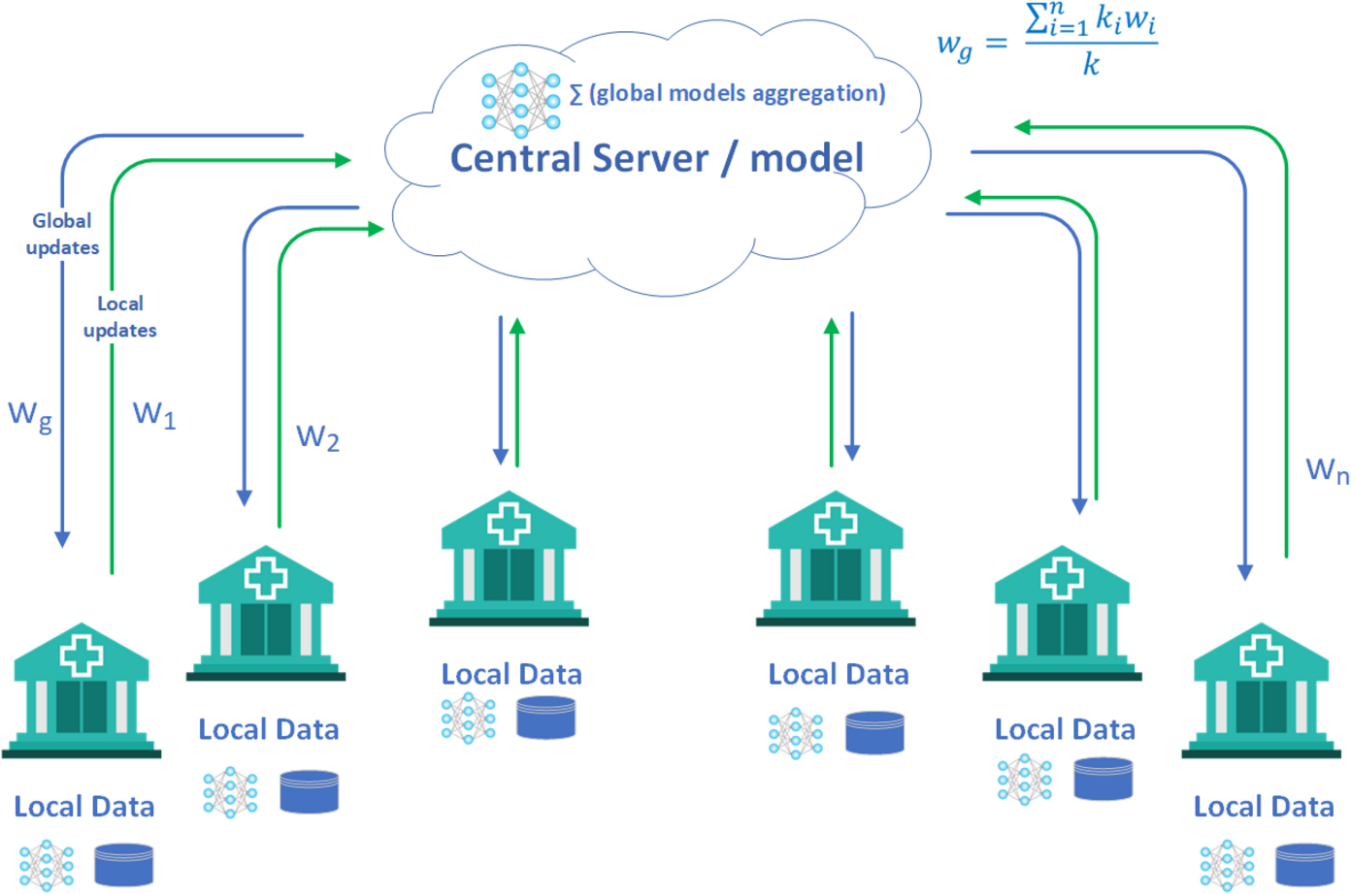
Federated learning is utilized for the prediction or categorization of medical records or diseases, prioritizing patient record confidentiality and minimizing network strain. Rather than sending raw data to a central server, an approach involving decentralized training is employed. Periodic communication between remote devices and a central server facilitates knowledge acquisition from a global model. Specific groups of computer systems conduct local training with diverse user data distributions, and the resultant updates are transmitted to the server. The server includes these updates and shares the improved global model with another set of devices. This process of iterative training persists until it reaches convergence or meets a pre-established stopping condition.

Federated learning shows significant potential in the field of healthcare data analytics. It offers promising prospects for both provider-oriented tasks, such as creating models to predict hospital readmission risks using patient Electronic Health Records (EHR) (Min, Yu & Wang, 2019), and consumer-focused applications, like detecting atrial fibrillation using electrocardiograms recorded by smartwatches (Perez et al., 2019). This methodology ensures that sensitive patient data remains confidential within local institutions or with individual consumers throughout the federated model learning process, thereby preserving patient privacy effectively. Federated learning involves the challenge of training a top-notch shared global model using a central server, drawing data from decentralized sources spread across a multitude of diverse clients (see Fig. 1).

### Challenges and opportunities in FL over wireless network.

In federated learning, local models are trained on end-devices and then combined on a central server. The global model is continuously sent back to end-devices until convergence is achieved. Federated learning relies on iterative interaction between end-devices and the aggregation server, necessitating efficient optimization schemes like FedAvg and FedProx. Subsequently, the aggregated weights (global model) are returned to the end-devices. Despite its benefits, federated learning encounters challenges such as privacy issues, resource optimization, incentive mechanisms, and statistical and system heterogeneity (McMahan et al., 2017). Malicious aggregation servers or end-devices could potentially extract sensitive information from others’ model updates, emphasizing the need for effective privacy preservation mechanisms. In summary, the discussion highlights the importance of addressing key questions regarding federated learning over wireless networks (Hong et al., 2021).

How can we design a federated optimization scheme to handle statistical and system heterogeneity effectively? How can we optimize computing and communication resources to shorten the global convergence time of federated learning? How do we enhance privacy in federated learning despite the existence of a malicious aggregation server?

To answer the above questions, we present a proposed network framework based on Wireless Backhaul Network.

### Aim of the study

Federated learning (FL) over wireless networks incorporates ML and Internet of Everything (IoE) applications, but there is room for improvement in resource allocation, security, and addressing statistical variations in FL aggregation algorithms. We have identified two key areas for improvement: (1) Enhancing wireless channels with terahertz spectrum, and (2) improving privacy in FL-based healthcare networks through a secure cross-silo solution using fixed wireless backhaul networks.

In this study, we explore a novel approach in the FL Briefing that leverages terahertz wireless channels and fixed wireless backhaul networks to address the challenges of resource efficiency and security. Additionally, we propose a design for implementing FL for healthcare using VPN-based wireless backhaul networks, including terahertz. This contribution is novel in the area of federated learning for healthcare improvement.

### Article structure

The article is structured as follows: It begins with an introduction and motivation for the study. “Proposed Solution” provides comprehensive details of the proposed solution and its key factors, while “Methodology” covers the methodology of the study. “Experiment Setup” and “Results and Discussion” explain the experiments, results, and discussion. Finally, the conclusion and future work of the study are presented.

## Proposed Solution

**Figure 2:**
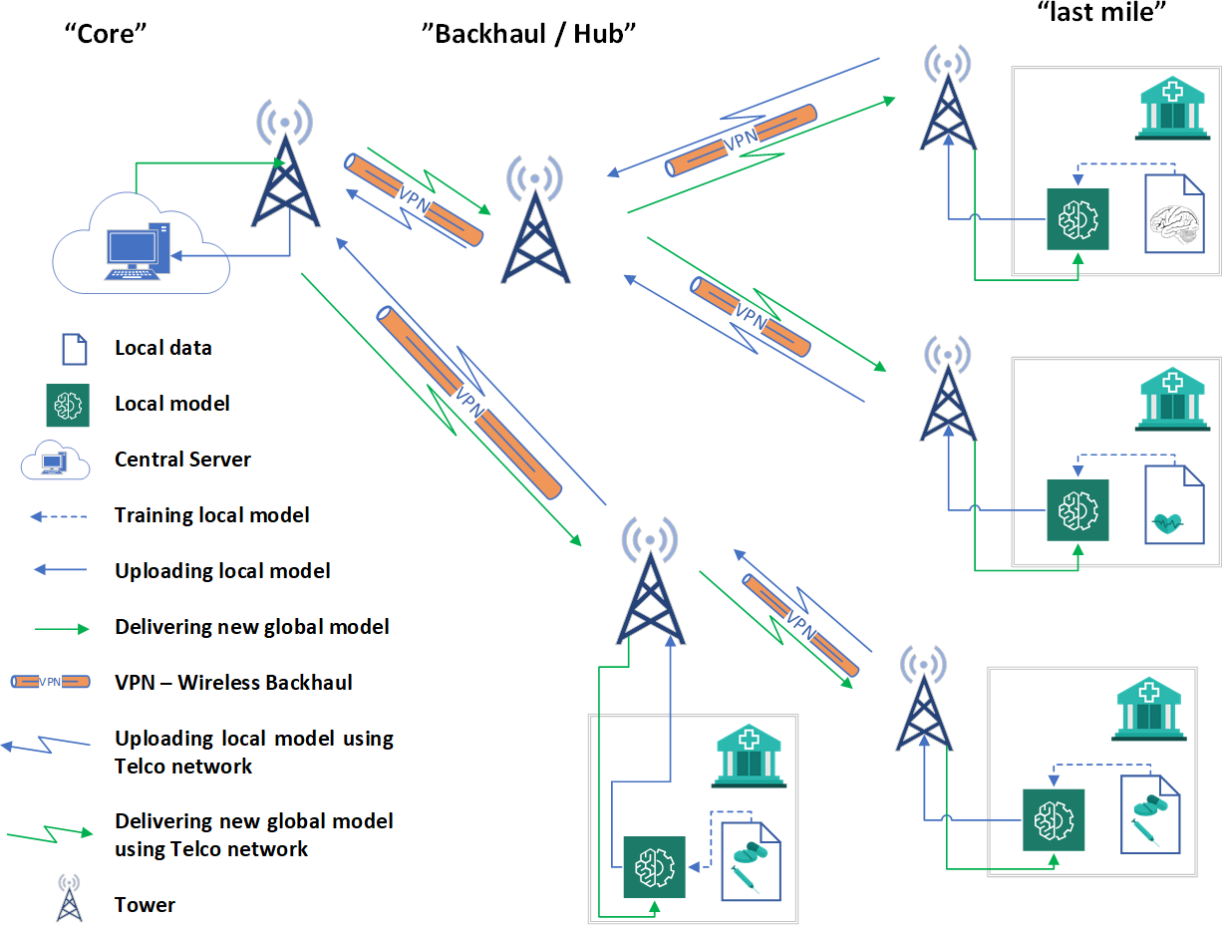
Proposed cross silo federated learning using VPN based wireless backhaul network (Mahmood et al., 2024).

The proposed framework comprises different technologies such as FL, wireless backhaul network, and virtual private network (VPN). Following our detailed discussions, we introduce the proposed framework depicted in Fig. 2 (Mahmood et al., 2024). Under this framework, each hospital functions as a client within a federated network, granting them independence to carry out specific tasks such as heart disease classification, MRI scan classification, medicine prediction, and various other medical analyses. In the proposed architecture, the federated network is implemented over a wireless backhaul network using the telecom network. Each site has a Radio Access Network (RAN) for user coverage and wireless backhaul links for data transfer between sites, with the final goal being the switching center. We use the backhaul link from these sites for our data transfer to the FL server. Let’s discuss this in more detail.

**Figure 3:**
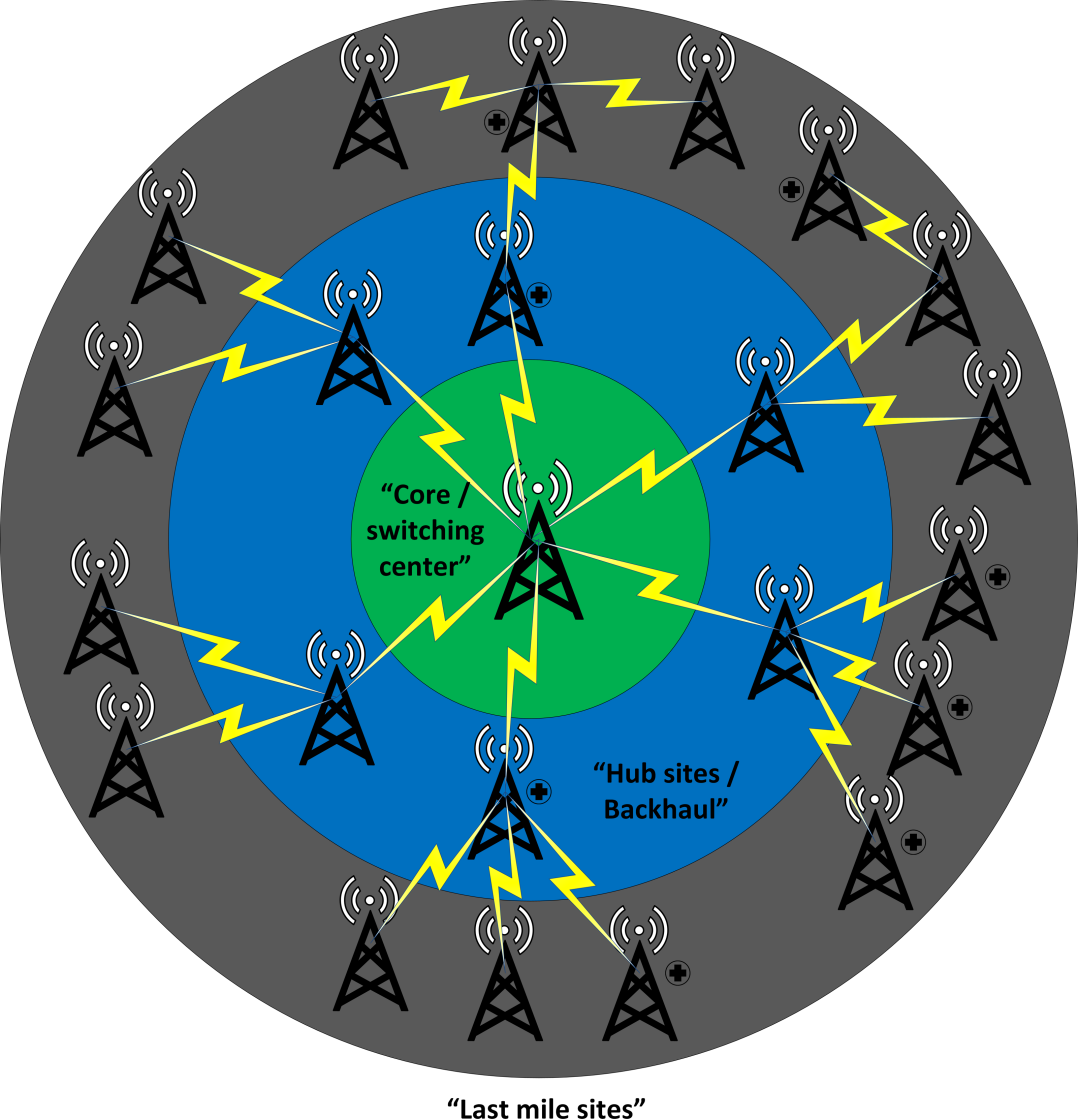
Proposed cross silo federated learning using VPN based wireless backhaul network.

Looking at how a mobile network is structured, we can identify three important parts that make it work: the last mile, the middle network, and the core network, as shown in Fig. 3. These parts work together to ensure the smooth operation of the mobile network, each playing its own unique and important role. The last mile site, vital in network paradigms (Parvez et al., 2018), is where users connect devices such as smartphones and smart gadgets to the wider network. This component ensures crucial network delivery and is indispensable in the mobile network ecosystem. Last mile 5G services deliver fast, low-latency, reliable connectivity using wireless technology, small cells, network slicing, and advanced antenna systems, benefiting various applications and industries to foster innovative connected experiences.

 Following the last mile is the regional network, which acts as a middle layer linking the last mile to the core network. It functions as a bridge, facilitating the flow of data and communication between the outer edges (last mile) and the central core. It is like connective tissue that ensures information can travel smoothly across the network, covering both urban and rural areas. The core network is the backbone of the mobile network, comprising optical and switching nodes across the nation. These nodes are resilient, scalable, and use advanced transport network protocols. Operating at the network’s core, it manages data flow, integrity, and essential network functions for modern mobile communication.

A cellular network’s success depends on strategic site selection, considering rental rates, civil engineering, RF optimization, marketing, profitability, and market dynamics. Assessments aim to use tall structures to lower tower costs and evaluate building strength to support equipment, towers, antennas, and an equipment room, ensuring resilience against equipment loads, seismic activity, and harsh weather (Mishra, 2018). We utilize high-rise buildings to install last mile sites and improve network coverage in crowded urban areas facing network congestion and signal issues. Elevated locations offer clear line-of-sight for mobile signals, enhancing signal quality and reliability. Installing infrastructure on tall buildings, such as malls, educational buildings, offices, and hospitals, increases network capacity, enabling more connections and better data traffic management.

Some of the sites deployed on hospital buildings and nearby locations are points of interest in this proposed architecture. The last mile sites and intermediate sites have a backhaul facility based on wireless backhaul links. We will use these links to transport the federated network traffic, which includes the model parameters. In Fig. 3, the sites marked with a hospital sign are used for this purpose and aim to transport the model parameters to the central server, where all parameters are aggregated and updated parameters are sent back to the users/clients. In this proposed scheme, the conventional traffic or normal backhaul traffic will not be disturbed, and a simple VPN architecture will be used. We will create a VPN on top of the conventional traffic, which will be a separate tunnel. This separate tunnel can be used to transmit and receive data for the federated network.

### Key factors of proposed architecture

In this section, we study the communication and security aspects of the proposed solution, focusing on several key considerations on which our proposed network is based and implemented:

• Private *versus* public network
• Licensed *versus* unlicensed channel
• Cross-silo *versus* cross-device
• Enhanced communication wireless channel

#### Private network

A private network ensures heightened security and a dedicated communication channel, separate from public networks. This reduces data breach risks and cyberattacks, allowing organizations full control over security protocols. The network’s private nature enables customized configurations, such as bandwidth allocation, network slicing, and application prioritization (Ezra et al., 2022). Private networks offer superior performance and reliability compared to public networks. They minimize network congestion by serving specific groups or organizations exclusively (Goyal, 2014).

The literature emphasizes the benefits of private networks over public ones, highlighting strengthened security measures and improved communication system quality, including bandwidth, integrity, and reliability. Private networks play a crucial role in protecting sensitive data and mitigating cyber threats, establishing a robust communication infrastructure (Abdulsalam & Hedabou, 2021).

#### Licensed channel

A wireless backhaul network in a licensed spectrum provides several advantages. It offers exclusive access to specific frequency bands, preventing interference from other users and allowing license holders to involve regulatory authorities in case of issues. Dedicated spectrum resources enable faster data transmission, outperforming unlicensed spectrum by delivering data ten times faster. This spectrum also allows greater flexibility in deploying cellular networks and ensures private access, reducing the risk of unauthorized data access and manipulation, critical in industries prioritizing data privacy. The literature here illustrates the benefits of licensed band security over unlicensed band and lists out the important aspects (Mumtaz et al., 2019; Grech & Eronen, 2005; Matinmikko et al., 2015; Cano et al., 2016).

#### Virtual private network (VPN)

VPNs prevent unauthorized access to private business correspondence and help reduce the environmental impact of business travel (Forbacha & Agwu, 2023). The VPN enhances security by integrating the public network into the private network and adds an extra layer of protection with another VPN within the private network. VPNs extend the capabilities of private networks to public networks, allowing devices to securely communicate as if they were directly connected to the private network. VPNs utilize different methods such as virtual point-to-point connections, dedicated links, tunneling protocols, and traffic encryption during setup. The discussion and literature show that VPNs can significantly add another layer of security for smooth communication (Ali, Hossain & Parvez, 2015; Odonkor et al., 2024; Akinsanya, Ekechi & Okeke, 2024; Iqbal & Riadi, 2019).

#### Cross-silo FL *vs.* cross-device FL

Cross-device FL involves a large number of clients contributing to the global model, utilizing extensive, dispersed data from the same application. Participating clients are decentralized entities, including smartphones, wearables, and edge devices. This approach poses challenges in managing transaction history logs due to the volume of clients and the use of unreliable networks, which can lead to random participation in training rounds and increased security risks.

**Figure 4:**
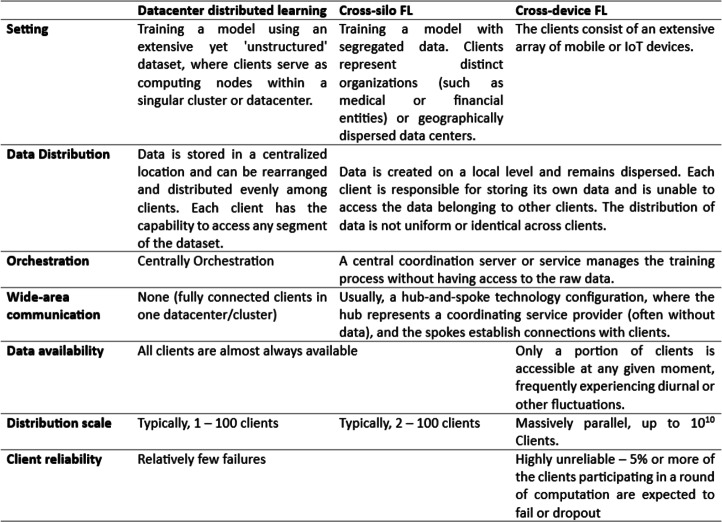
Comparison of cross-silo and cross-device settings.

In contrast, cross-silo federated learning typically involves 2 to 1,000 devices within a trusted network, consistently available for training rounds. This approach offers more flexibility than cross-device FL and is commonly used in organizational or group settings for training models with confidential data. Essential techniques such as client selection and incentive designs facilitate this form of federated learning (Yu et al., 2020). The comparison of both data availability approaches is explained in Fig. 4.

#### Enhance wireless communication channel

We are dealing with a private network, specifically a wireless backhaul network. This network operates only under licensed spectrums such as microwave, mmWave, and the newly studied spectrum, terahertz (THz). In our previous study We propose terahertz-based wireless backhaul as integral to 6G due to its numerous benefits: significantly higher bandwidth in the terabits per second (Tbps) range, exceptional user data rates up to 100 Gbps, ultra-low latency under 0.1 ms, improved spectrum efficiency, and a tenfold increase in bandwidth compared to traditional solutions. This technology promises unprecedented speeds and is ideal for next-gen applications and services. The suggested design is versatile and perfectly suited for various wireless backhaul networks operating at frequencies starting from 38 GHz and beyond. Its capabilities, combined with the benefits of terahertz technology, establish it as a promising and forward-looking solution to meet the changing connectivity requirements of the 6G era (Mahmood et al., 2024).

## Methodology

**Figure 5:**
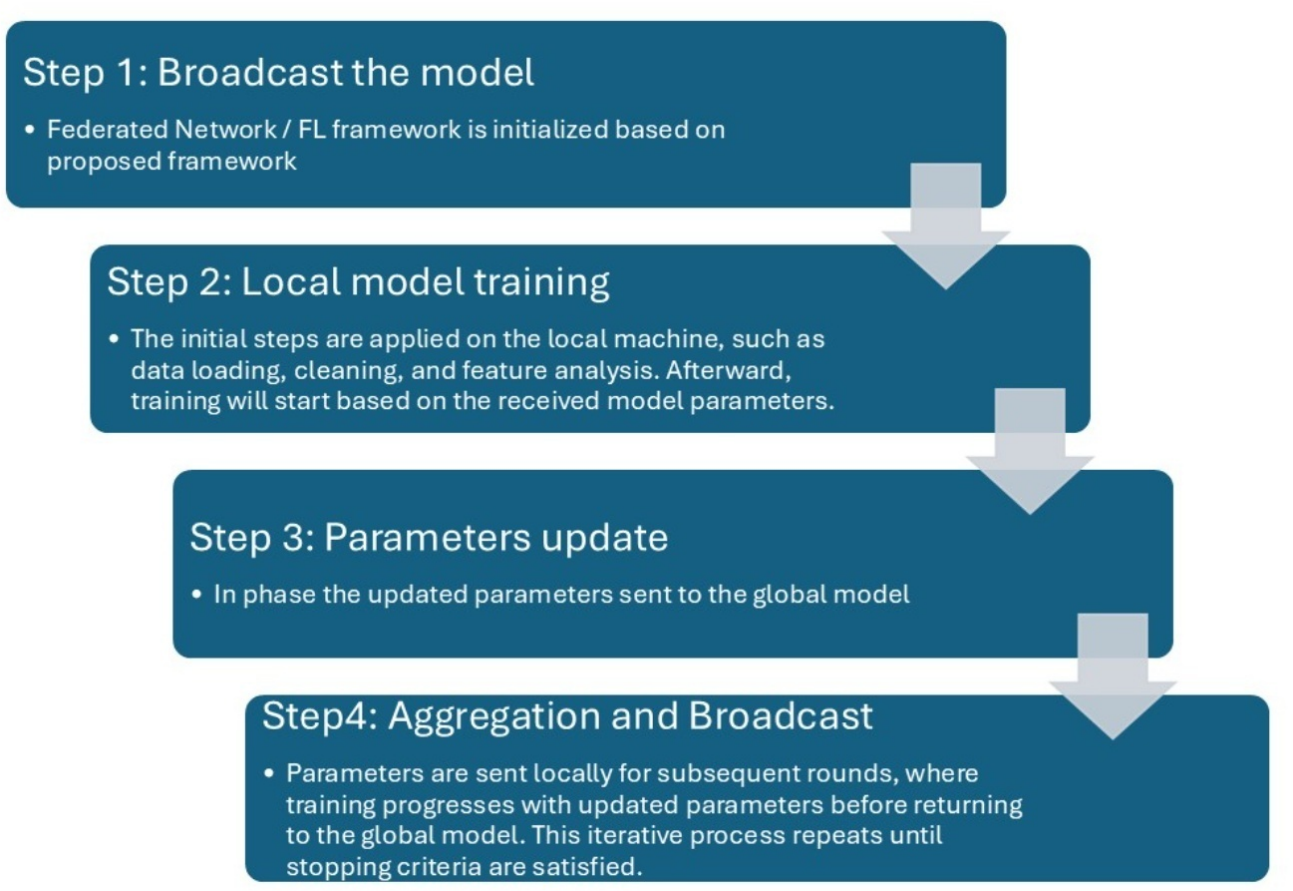
Brief methodology of experiments/structure.

In this section, we have explained the overall methodology as depicted in Fig. 5. The remaining article is structured based on the methodology discussed in this section. In our upcoming analysis, we intend to focus on hand, foot, and mouth disease (HFMD) classification using biosensor-extracted data. We will assess the performance of our proposed framework in this context. To establish a validation benchmark, we compared these analyses with the results obtained through centralized approaches currently available, as comprehensively explained in Mahmood et al. (2022). The results from Mahmood et al. (2022) are now used for further comparative analysis, enabling us to draw conclusions about the achievements of our proposed framework and identify areas for potential improvement.

### Flow of experiments

**Figure 6:**
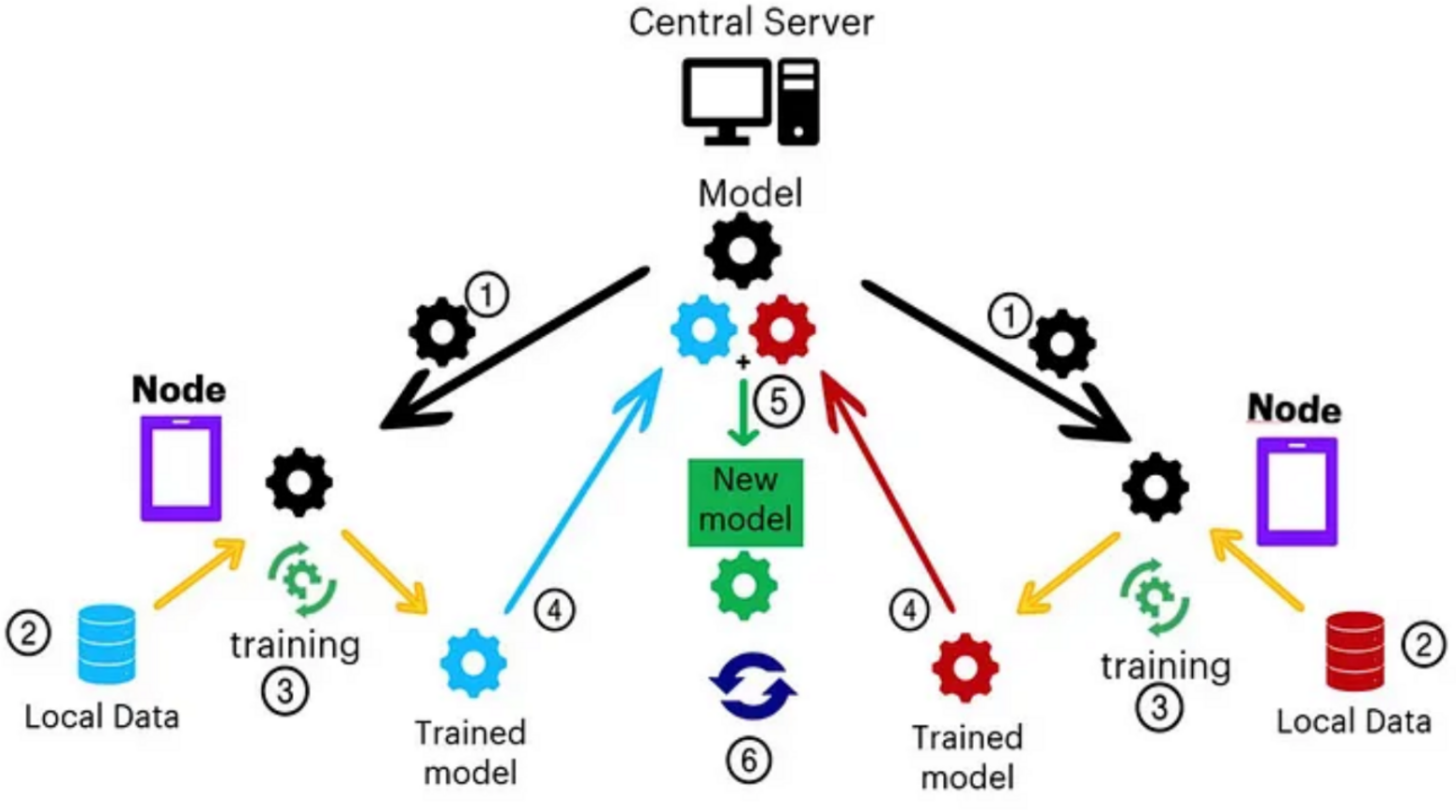
Steps in training a model using the FL framework, with nodes referring to hospitals or clinics (Fachola et al., 2023).

In this section, we explain the flow of the experiment and how federated ML performs the classification of signals. In Fig. 6, the stepwise approach for federated ML is illustrated. In Step 1, the central server sends the latest model parameters to the nodes. Step 2 involves data collection at each node. In Step 3, each local model is trained based on the latest parameters. Step 4 consists of communicating the updated model parameters back to the global model. In Step 5, the updates from each model are combined to retrain the global model, resulting in a new model. Finally, Step 6 restarts the process from Step 1.

### Machine learning for classification: state of the art

Machine learning a broad topic in the field of artificial intelligence (AI) that involves developing algorithms and models that enable computers to learn from and make predictions or decisions based on data. Machine learning has the potential to revolutionize various industries by making predictions, automating tasks, and extracting valuable insights from data In the realm of machine learning, numerous situations present difficulties when it comes to transferring data to a central server for model training. To tackle these issues, an alternative approach is to employ distributed machine learning, also detailed in Verbraeken’s survey (Verbraeken et al., 2020).

#### Artificial neural network (ANN)

**Figure 7:**
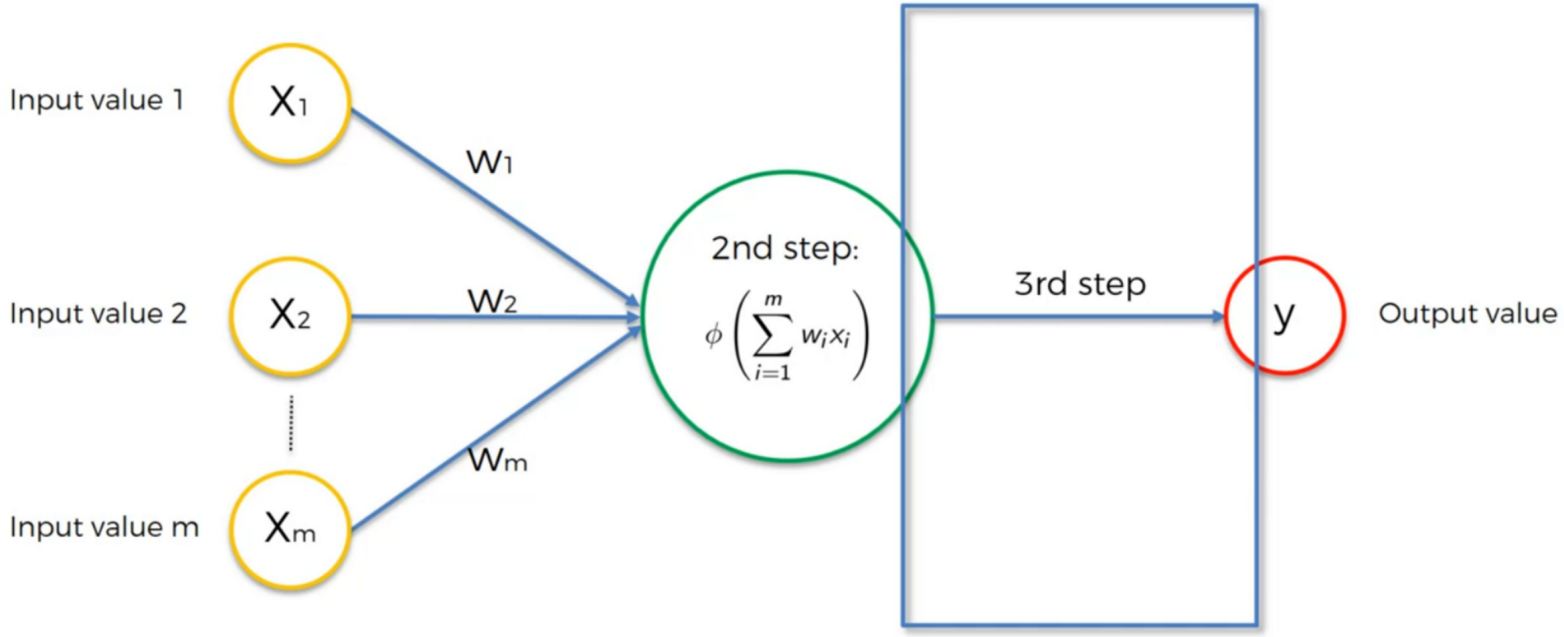
A basic neural network is portrayed, comprising a three-input input layer and lacking hidden layers. The output layer receives the combined inputs and weights, undergoes activation by any chosen function, and then progresses to produce the final output.

**Figure 8:**
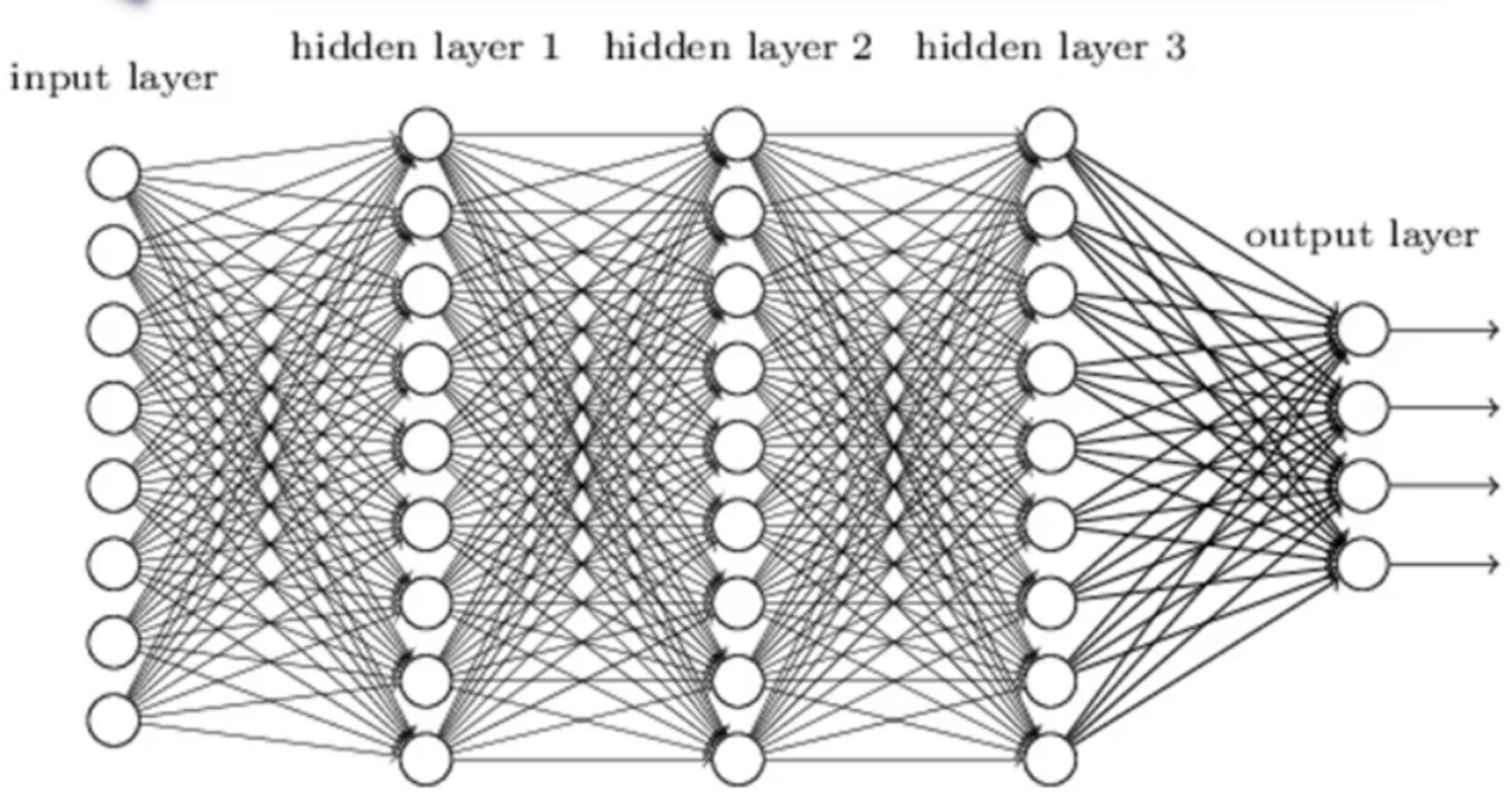
The depiction showcases a neural network characterized by a solitary input layer capable of handling multiple inputs. This network is distinguished by three hidden layers and four outputs at the output layer.

Inspired by the human brain, a neural network creates meaningful features from an initial signal using interconnected layers of units. The first layer accepts diverse inputs, and the last layer acts as a classifier. Intermediate layers transform inputs into valuable features, enabling the network to acquire robust characteristics for classification as presented in Figs. 7 and 8. Training employs stochastic gradient descent, removing the necessity for manual feature selection. This method, especially advantageous for classification, has resulted in highly efficient image and non-image classification systems based on neural networks (Bishop, 2006).

#### Neural network—federated learning

Distributed machine learning involves training numerous models across servers that are located in different geographical locations, and these models are later combined to create a unified machine learning model. Federated learning is a key modern-day solution for addressing the challenges of training a machine learning model on extremely large datasets, such as astronomical data, which include substantial computational energy consumption and extended training durations (Li et al., 2020). It is a machine learning method enabling training models on decentralized devices like smartphones, IoT devices, or local servers, without transferring data to a central server. In contrast to traditional machine learning, where data is usually gathered and sent to a central server for model training. However, in federated learning, the model is sent to the data sources, and model updates are performed locally on these devices. In this way, FL earns many advantages over other approaches such as privacy preservation, reduced data transfer, decentralization and model customization.

In recent times, there has been a notable increase in attention towards the field of healthcare data analytics. This heightened interest is attributed to the growing availability of healthcare data from diverse sources, such as clinical institutions, individual patients, insurance firms, and pharmaceutical companies. This influx of data presents an unparalleled opportunity to develop computational methods aimed at extracting valuable insights from the data. These insights, driven by data, have the potential to enhance the quality of healthcare delivery significantly (Wang & Preininger, 2019).

In applying federated learning referred to Fig. 1, to classify diseases on computer systems, we prioritize patient data privacy and network efficiency. Instead of sending raw data to a central server, a decentralized approach is adopted. Remote devices periodically communicate with a central server to obtain a global model. During each communication round, a specific subset of devices conducts local training on their distinct patient data, sending updates to the server. The server adjusts the global model and transmits it to another subset of devices. This iterative process continues until convergence or a predefined stopping criterion is met.

### Description of datasets

The data is in the form of a signal extracted from a biofunctionalized SPR-TFBG sensor. To clarify and simplify, we explained how the biofunctionalized SPR-TFBG sensor works. The primary function of the SPR-TFBG biosensor is to differentiate the spectral details between a virus-free solution and a virus-containing solution. The SPR mode’s spectral responses vary with different concentrations of the EV-A71 virus in both clean solutions and solutions with varying levels of impurities. Readers are encouraged to review the additional information on the development and experiments of the SPR-TFBG biosensor found in Udos et al. (2021).

**Figure 9:**
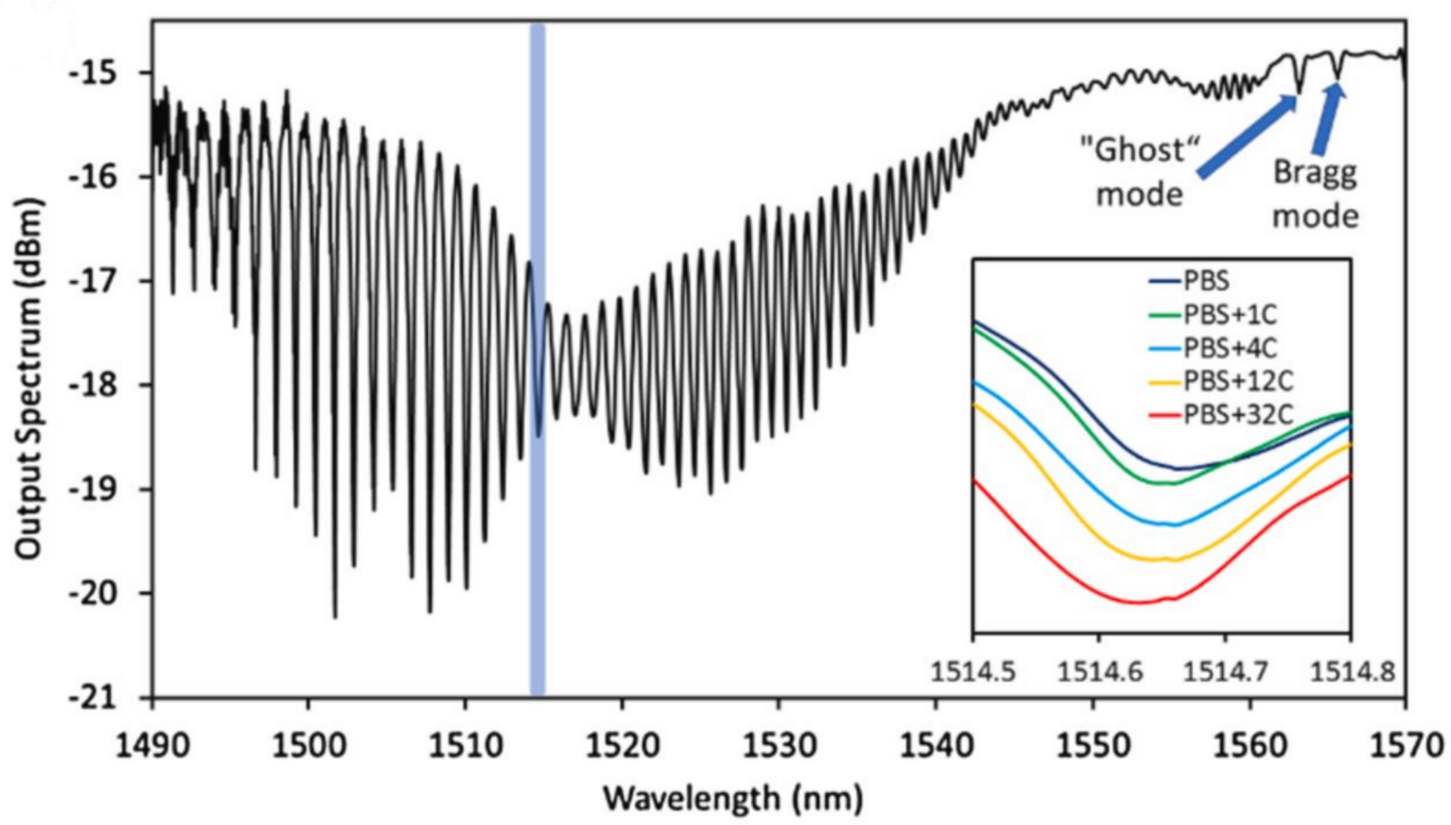
The PBS-immersed biofunctionalized SPR-TFBG exhibits a transmission spectrum in the P-polarized mode. The selected SPR mode (1514.6 nm) for the virus test is emphasized through the shaded region. Indications of the Bragg mode and ghost mode are depicted by blue arrows. The inset illustrates the SPR mode’s spectral responses to diverse concentrations of the EV-A71 virus (Udos et al., 2021).

The horizontal axis corresponds to the wavelength, and the vertical axis represents the amplitude of each wavelength in decibels (dBm). Figure 9 illustrates the relationship between wavelength and amplitude for each solution. Referring to the raw data, the signal waveform spans from 1,490 nm to 1,570 nm, with the *y*-axis representing the output spectrum in dBm, ranging from −21 to −15. Each signal comprises 4,000 individual features.

In other words, one sample or single patient data has 4,000 individual features based on the waveform, and their values vary from −21 to −15. The matrix of the complete dataset for all impurities and a clean solution has a dimension of n×4000, where 4,000 are the number of features and n is the number of samples. In the next step of dimension reduction, the region of interest (ROI) is applied. The region of interest ranges from 1,510 nm to 1,540 nm, meaning the ROI has 1,496 features of different wavelengths, and the dimension has now been reduced to n×1,496. There are five different classes available in the datasets based on expert opinion and analysis: PBS, 02 µL, 08 µL, 32 µL, and 128 µL. PBS refers to the clean solution, meaning there are no virus strains present, while 02 µL to 128 µL refer to impure solutions with varying virus intensities and types. The sample dataset is presented in Table 1. There are a total of four clients taking part in the classification process. Client 1 contributes 598 samples, Client 2 contributes 287 samples, Client 3 has 275 samples, and Client 4 has 162 samples. There are 264 samples tested in each solution: PBS, 02 µL, 08 µL, 32 µL, and 128 µL. The total number of tested samples is 1,320 when all the samples from all the clients are consolidated.

**Table 1:** All solutions with labels written in Target col after feature reduction based on ROI.

**Features**	**1510.1**	**1510.12**	. . .	**1540**	**TARGET**
Sample-1	−21.5246	−21.5343	. . .	−18.9976	2UL
Sample-2	−21.5359	−21.5545	. . .	−18.9935	2UL
Sample-140	−21.4907	−21.5165	. . .	−18.9346	8UL
Sample-141	−21.5351	−21.5069	. . .	−19.0065	8UL
Sample-265	−21.4415	−21.4617	. . .	−18.9637	32UL
Sample-266	−21.3745	−21.3842	. . .	−18.9193	32UL
Sample-399	−21.5416	−21.5569	. . .	−19.0573	128UL
Sample-400	−21.6037	−21.6166	. . .	−19.0444	128UL
Sample-531	−21.4818	−21.4609	. . .	−18.9209	0UL
Sample-532	−21.5174	−21.5206	. . .	−19.0048	0UL

The merged dataset for all the impurities and a clean solution is presented in Table 1. In the final dataset, each wavelength is treated as the individual feature of the samples. In the final image of the dataset presented in Table 2, there are around 4,000 features for every sample. Each sample is labeled as 0 ul, 02 ul, 08 ul, 32 ul, and 128 ul.

**Table 2:** Statistical characteristics used for features (Mahmood et al., 2022).

**Statistical characteristics**	**Formula**
Mean	$(\mu )= \frac{1}{n} {\mathop{\sum }\nolimits }_{i=1}^{n}{x}_{i}$
Root mean square (RMS)	$RMS=\sqrt{ \frac{1}{n} {\mathop{\sum }\nolimits }_{i=1}^{n}({x}_{i})^{2}}$
Variance	${\sigma }^{2}= \frac{1}{n} {\mathop{\sum }\nolimits }_{i=1}^{n}({x}_{i}-\mu )^{2}$
Standard deviation	$\sigma =\sqrt{ \frac{1}{n} {\mathop{\sum }\nolimits }_{i=1}^{n}({x}_{i}-\mu )^{2}}$
Skewness	$ \frac{1}{n} {\mathop{\sum }\nolimits }_{i=1}^{n}( \frac{{x}_{i}-\mu }{\sigma } )^{3}$
Kurtosis	$kurtosis= \frac{1}{n} {\mathop{\sum }\nolimits }_{i=1}^{n}( \frac{{x}_{i}-\mu }{\sigma } )^{4}$
Crest Factor	$CF= \frac{Max({|}x{|})}{RMS} $
Impulse Factor	$IF= \frac{Max({|}x{|})}{ \frac{1}{n} {\mathop{\sum }\nolimits }_{i=1}^{n}{|}x(t){|}} $
Shape Factor	$SF= \frac{RMS}{ \frac{1}{n} {\mathop{\sum }\nolimits }_{i=1}^{n}{|}x(t){|}} $
Range	*Range* = *Max*(*x*) − *Min*(*x*)

### Feature analysis

The section is divided into feature extraction and feature engineering for both centralized and federated ML approaches. In each part, we explained the technique that is used, and then their results for both approaches are presented separately.

#### Feature extraction

The utilization of raw signals by the classifier might lead to bias or influence its performance due to the abundance of features. Table 1 displays the existence of 1496 features originating from diverse wavelengths within the region of interest (ROI). In the initial step of feature extraction, a simple bandpass filter was employed on the wavelength spectrum encompassing the ROI. This specific range extends from 1,510 nm to 1,540 nm, as detailed in Table 1.

#### Feature engineering

Engaging in feature engineering involves significant computational demands, often giving rise to the creation of numerous sets of features. The presence of these numerous feature sets can give rise to a range of challenges. Some of these difficulties encompass the usage of computationally resource-intensive models, the risk of model overfitting, a decline in model effectiveness, issues with multi-collinearity, and the potential drawbacks of dealing with high dimensions. To address these potential problems, we have implemented techniques for feature reduction, guided by the statistical characteristics of the signals. This process aims to produce a condensed set of features that can lead to heightened accuracy, thus markedly improving the performance of classification. The selected attributes for this process include mean, SD, variance, RMS, kurtosis, skewness, crest, shape, range and impulse, all derived from their corresponding statistical characteristics (Kuhn & Johnson, 2019).

The statistical characteristics displayed in Table 2 are explained in detail in our previous study, including their definitions and descriptions. Here, we describe their mathematical formulas. Later, the features extracted through statistical properties are displayed in Table 3 refereed by our previous study (Mahmood et al., 2022). Following the application of feature engineering guided by these statistical properties, the dataset’s dimension is reduced from 1,496 to 10.

**Table 3:** Statistical characteristics derived from the combined datasets of all solutions within the ROI.

**Features**	**IF**	**SF**	**CF**	**RMS**	**Kur**	**Skew**	**Var**	**SD**	**Mean**	**Range**	**TARGET**
sample-1	0.911	1.002	0.910	20.661	−0.370	−0.634	1.576	1.255	−20.622	5.190	2UL
sample-2	0.911	1.002	0.909	20.669	−0.397	−0.625	1.599	1.265	−20.630	5.216	2UL
sample-135	0.912	1.002	0.911	20.640	−0.317	−0.653	1.562	1.250	−20.603	5.189	8UL
sample-136	0.911	1.002	0.910	20.652	−0.351	−0.647	1.591	1.261	−20.614	5.206	8UL
sample-265	0.912	1.002	0.910	20.601	−0.363	−0.633	1.607	1.268	−20.562	5.230	32UL
sample-266	0.911	1.002	0.910	20.594	−0.360	−0.637	1.599	1.264	−20.555	5.182	32UL
sample-399	0.912	1.002	0.911	20.714	−0.354	−0.642	1.585	1.259	−20.676	5.184	128UL
sample-400	0.914	1.002	0.913	20.710	−0.369	−0.630	1.580	1.257	−20.672	5.136	128UL
sample-531	0.910	1.002	0.909	20.631	−0.391	−0.611	1.578	1.256	−20.593	5.189	0UL
sample-532	0.912	1.002	0.910	20.626	−0.372	−0.629	1.579	1.257	−20.588	5.165	0UL

#### Feature selection

**Figure 10:**
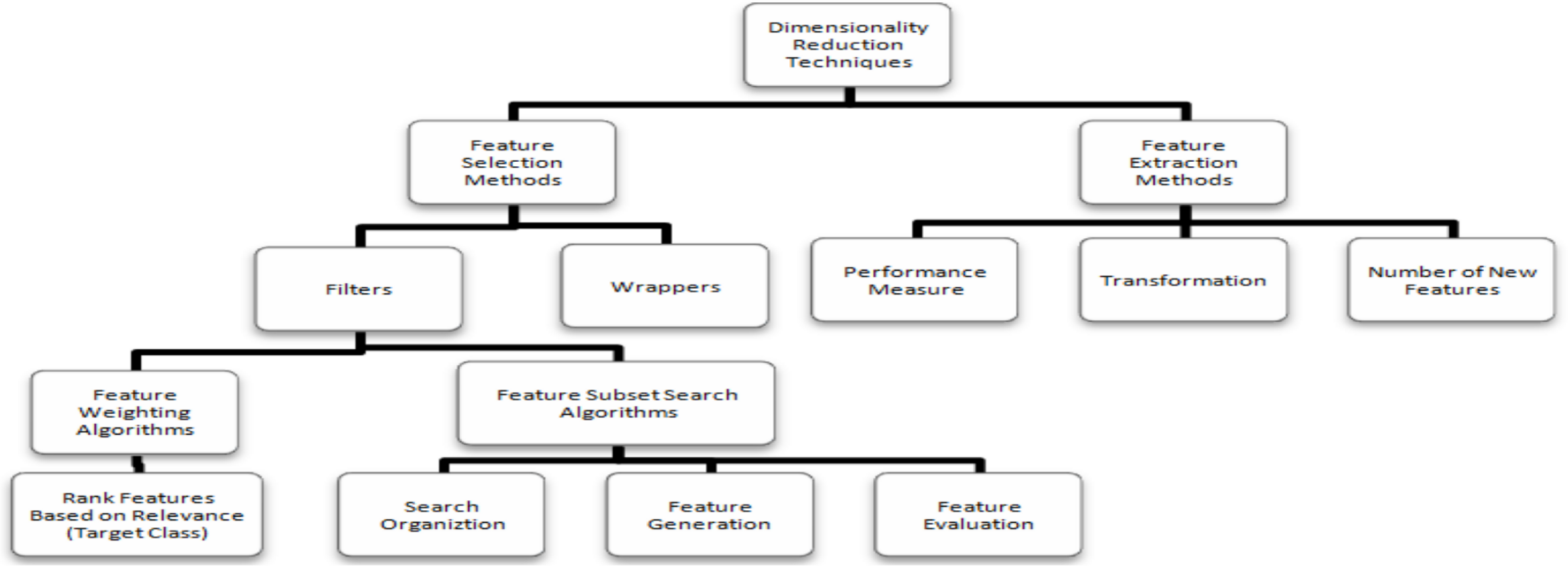
Feature selection techniques (Khalid, Khalil & Nasreen, 2014).

Following feature extraction, the subsequent stage involves feature selection. Numerous techniques exist to tackle the issue of diminishing irrelevant and redundant features that can complicate a demanding task. Generally, the feature selection methods fall into categories such as filters, wrappers, and embedded methods, as depicted in Fig. 10.

##### Filter methods —mutual information.

Filter techniques utilize variable ranking strategies as the fundamental basis for selecting variables by means of ordering. The selection of ranking methods stems from their simplicity and proven effectiveness within practical contexts. A fitting ranking criterion is applied to assign scores to the variables, and subsequently, a threshold is employed to exclude variables that do not meet the threshold. Ranking methods operate akin to filter methods, given that they are implemented prior to classification, with the aim of sieving out less pertinent variables. A fundamental quality of a distinctive feature lies in its capacity to encompass valuable insights concerning the various classes inherent in the data. This attribute can be defined as feature relevance, offering a metric for assessing the feature’s capability to distinguish among the diverse classes present. In this investigation, we have chosen mutual information as the filter approach to designate the selected features (Chandrashekar & Sahin, 2014).

Utilizing the concept of mutual information (MI) introduced by Deng, Luo & Yu (2014), a term weighting scheme was developed. For a given term ti and a document set Dc (comprising documents within a specific category), the MI between them is computed using the subsequent formula:

$MI({t}_{i},{D}^{c})=log \frac{P({t}_{i},{D}^{c})}{P({t}_{i})xP({D}^{c})} $

**Figure 11:**
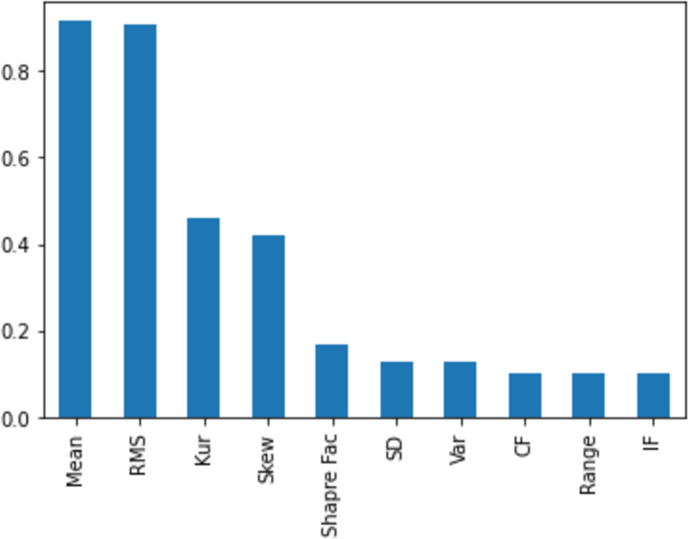
The feature selection on the centralized dataset, comprising a total of 1,320 samples, is presented here with scores based on the MI method, ranging from a maximum to a minimum. It is evident that the feature ‘mean’ attains the highest score, while the feature ‘IF’ receives the lowest score. This information can be subsequently utilized for feature selection.

**Figure 12:**
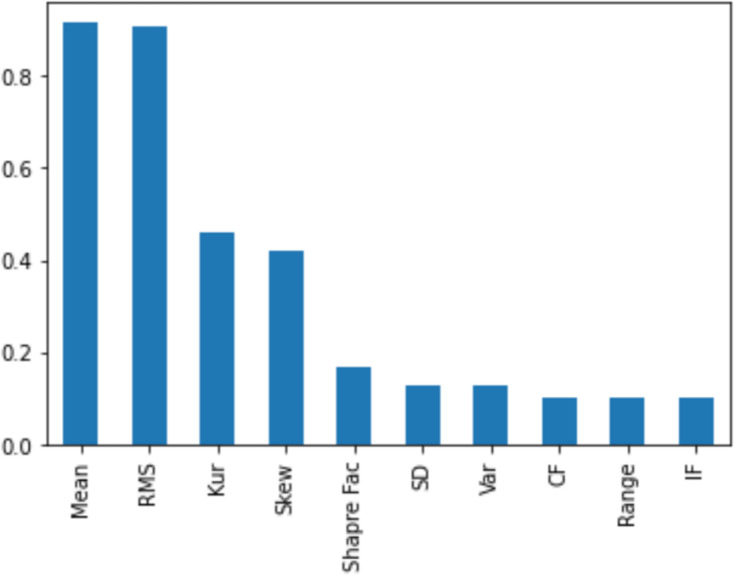
The feature selection on the distributed dataset, based on four clients where Client 1 has 598 samples. In the left image, the MI score of Client 1 is displayed, revealing that the feature ‘mean’ achieves the highest score, while the feature ’SD’ obtains the lowest score.

**Figure 13:**
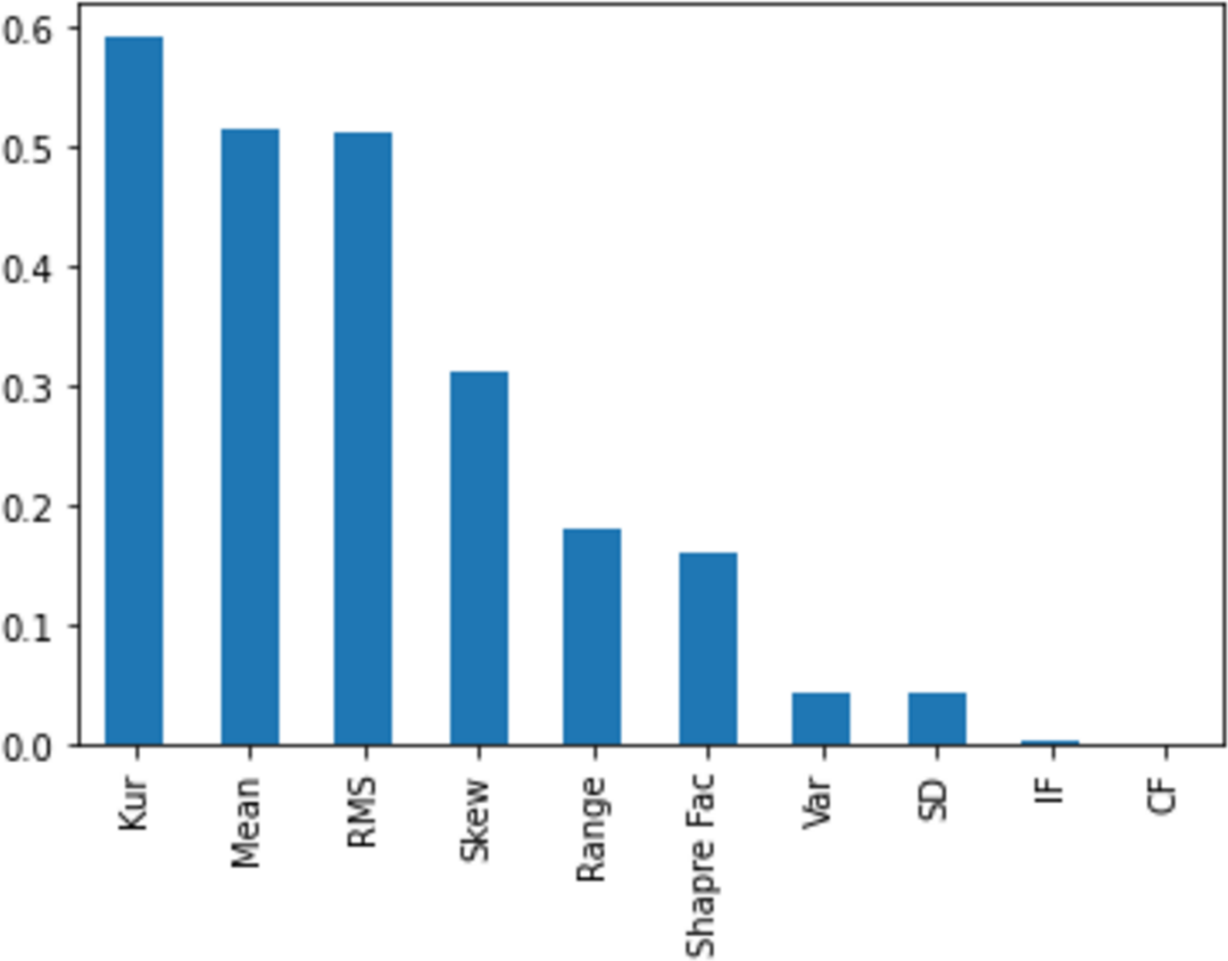
Similarly, the right-side image shows the feature selection on Client 2, comprising 287 samples, with the highest score assigned to the feature ‘kurtosis’ and the lowest to ‘CF’.

**Figure 14:**
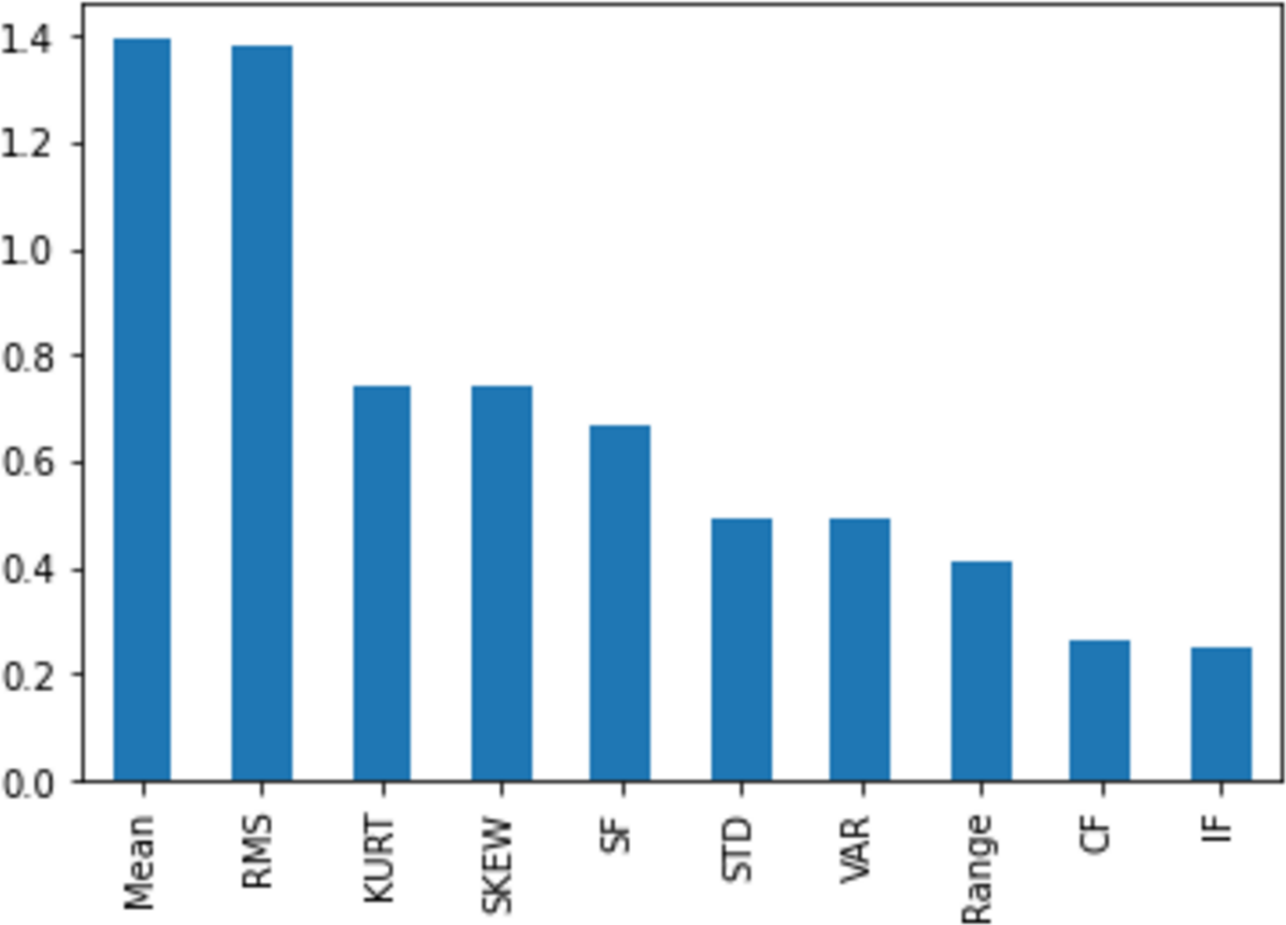
The feature selection on the distributed dataset, based on four clients, where Client 3 has 275 samples, is presented. The MI score of Client 3 is displayed, revealing that the feature ‘mean’ achieves the highest score, while the feature ‘IF’ obtains the lowest score.

**Figure 15:**
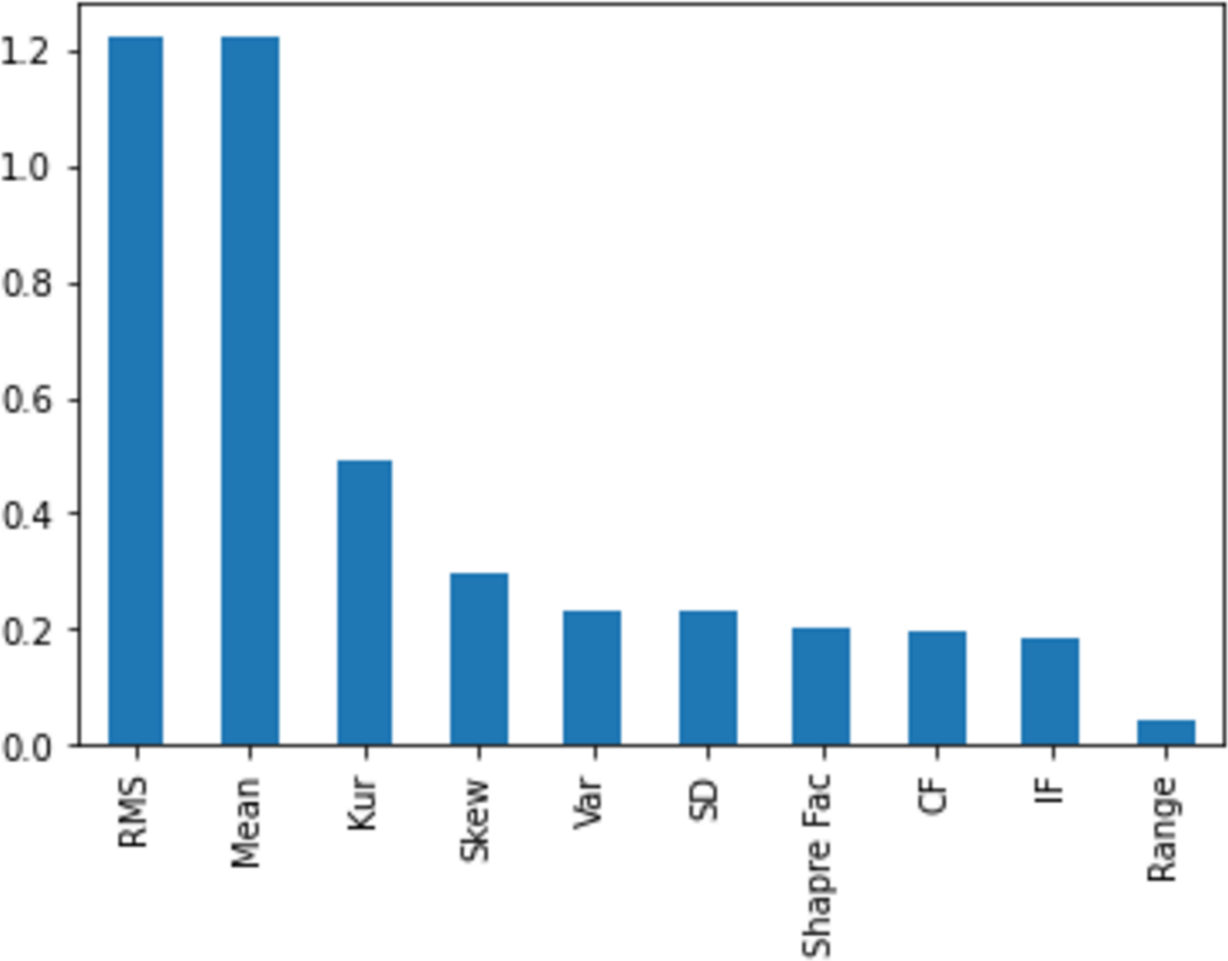
Image shows the feature selection on Client 4, comprising 162 samples, with the highest score assigned to the feature ‘RMS’ and the lowest to ‘Range’.

Figures 11, 12, 13, 14 and 15 illustrate the feature set along with their MI Score using the method for both centralized ML and federated ML. From the selected features, we targeted the top 80% and compare the results with the sequential feature selection method. These selected feature sets are further employed for classification purposes. In the classification phase, we selected all five feature sets and run classifier, evaluating the performance and accuracy based on the established criteria.

The top eight out of 10 feature sets used for centralized machine learning classification and for distributed clients in federated networks refer to specific attributes or characteristics of data selected based on mutual information, as presented in Table 4.

### Performance evaluation

The classification process serves as the ultimate and crucial stage for the identification and categorization of both pure and impure solutions, each characterized by varying degrees of impurities. According to the established methodologies, the features identified and listed in Table 4 *via* employing MI will be employed to classify the signals. Consequently, the signals will be grouped into virus-free or virus-present categories, depending on the impurity level. The dataset was divided into separate testing and training sets. In order to assess the results obtained across different classifiers, the following metrics were employed:

• True positives (TP) correspond to cases where the expert-annotated positive class coincides with the model’s positive class prediction.
• False positives (FP) represent instances where the expert-annotated negative class is erroneously predicted by the model as the positive class.
• True negatives (TN) indicate instances where the expert-annotated negative class is accurately predicted as the negative class by the model.
• False negatives (FN) denote cases where the expert-annotated positive class is incorrectly classified by the model as the negative class. These performance metrics were employed in this study to assess the outcomes achieved through various classifiers.:

(1) \begin{eqnarray*}Accuracy= \frac{TP+TN}{TP+FP+FN+TN} \end{eqnarray*}

(2) \begin{eqnarray*}Sensitivity= \frac{TP}{FN+TP} \end{eqnarray*}

(3) \begin{eqnarray*}Precision= \frac{TP}{TP+FP} \end{eqnarray*}

(4) \begin{eqnarray*}F1score=2\ast \frac{precision\ast recall}{precision+recall} .\end{eqnarray*}

## Experiment Setup

### Experiment with neural network—centralized ML

This study implemented a neural network using Python and the Keras library, which is a user-friendly high-level Python library designed for deep learning. Keras seamlessly integrates with TensorFlow (or Theano or CNTK), focusing on essential deep learning concepts such as creating neural network layers while managing tensor details. TensorFlow (or Theano or CNTK) acts as the backend for Keras. By utilizing Keras in deep learning applications, practitioners can avoid dealing directly with the complexities of TensorFlow (or Theano or CNTK). Keras offers two main frameworks: the sequential API, which arranges layers in a linear sequence, and the functional API. The sequential model represents a straightforward stack of layers.

#### Model architecture

##### Input layer.

In our experimentation, we utilized feature sets detailed in Table 3. Initially, we applied MI with an eight-feature set, selecting features based on their relevance using this information-theoretic measure. This diverse approach to feature engineering allows our neural network to adapt and optimize performance according to each set’s unique characteristics.

##### Hidden layers.

In building our neural network, we intentionally opted to use two hidden layers for specific feature set methodologies. Each hidden layer consists of 15 nodes, carefully selected to enhance the extraction of pertinent patterns and relationships within the input data. This setup is customized to suit the distinctive attributes and informational richness of the Mutual Information features.

##### Output layer.

In our classification task focused on virus stages, we predict five distinct classes: 0 UL (PBS), 2 UL, 8 UL, 32 UL, and 128 UL. The output layer of our neural network is configured with five neurons, each corresponding to one of these classes. This design allows the model to predict the likelihood of an input instance belonging to any of these stages, ensuring precise classification tailored to the nuances of virus stages.

##### Activation functions.

In designing this neural network architecture, we’ve integrated two distinct activation functions with specific roles. The rectified linear unit (ReLU) is chosen for the hidden layers to introduce crucial non-linearity, enabling effective capture of complex patterns and relationships in input data. ReLU also mitigates the vanishing gradient problem, enhancing learning efficiency during training. Softmax is chosen for the output layer to facilitate multi-class classification tasks by transforming the network’s outputs into a probability distribution across multiple classes. This feature is particularly advantageous for categorizing input instances into distinct categories. The deliberate use of ReLU for hidden layers and Softmax for the output layer demonstrates a thoughtful approach, optimizing the model’s performance for its specific task.

**Table 4:** Features set used for classification.

**Dataset & Approach**	**Feature selection**	**Feature set**
Consolidated dataset for centralized ML	MI with Top 80%	(‘Mean’, ‘RMS’, ‘Kur’, ‘Skew’, ‘Shape Fac’, ‘SD’, ‘Var’, ‘CF’)
Client 1 for federated ML	MI with Top 80%	(‘Mean’, ‘RMS’, ‘Kur’, ‘Skew’, ‘CF’, ‘IF’, ‘Range’, ‘Shape Fac’)
Client 2 for federated ML	MI with Top 80%	(‘Kur’, ‘Mean’, ‘RMS’, ‘Skew’, ‘Range’, ‘Shape Fac’, ‘Var’, ‘SD’)
Client 3 for federated ML	MI with Top 80%	(‘Mean’, ‘RMS’, ‘Kur’, ‘Skew’, ‘Shape Fac’, ‘SD’, ‘Var’, ‘Range’)
Client 4 for federated ML	MI with Top 80%	(‘RMS’, ‘Mean’, ‘Kur’, ‘Skew’, ‘Var’, ‘SD’, ‘Shape Fac’, ‘CF’)

#### Experiment results

**Figure 16:**
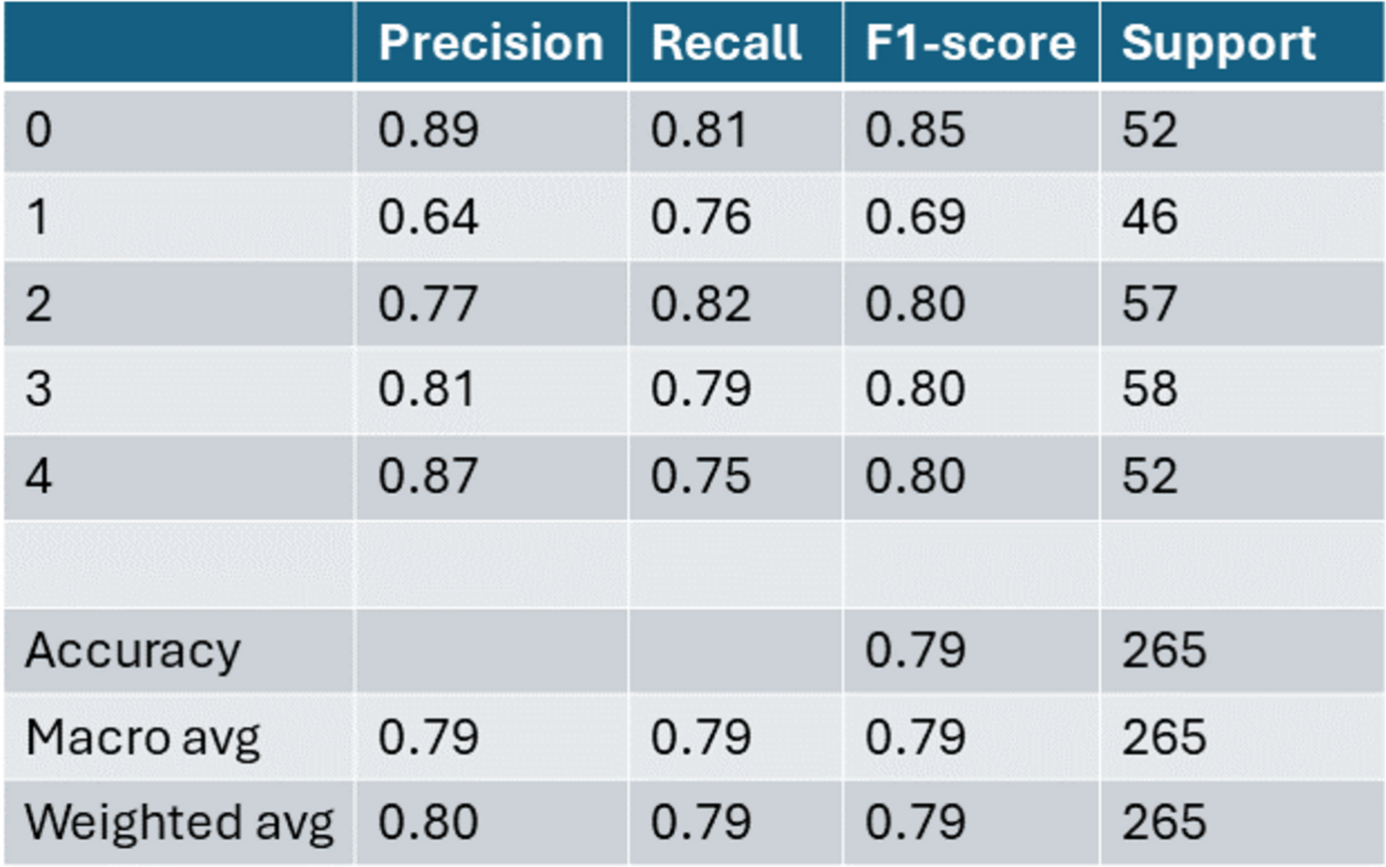
Performance analysis of neural network using mutual information features set achieved 80% weighted accuracy.

Referring to Table 1 and ‘Artificial neural network (ANN)’, our consolidated dataset consists of a total of 1,320 samples, each characterized by eight distinct features, as described earlier. The centralized machine learning architecture previously described is applied to this dataset, and the results achieved are presented in Fig. 16. In this scenario, the dataset is split into training and testing sets using a 70–30 percent ratio, with 70 percent allocated for training and the remaining 30 percent for testing.

### Federated ML

**Figure 17:**
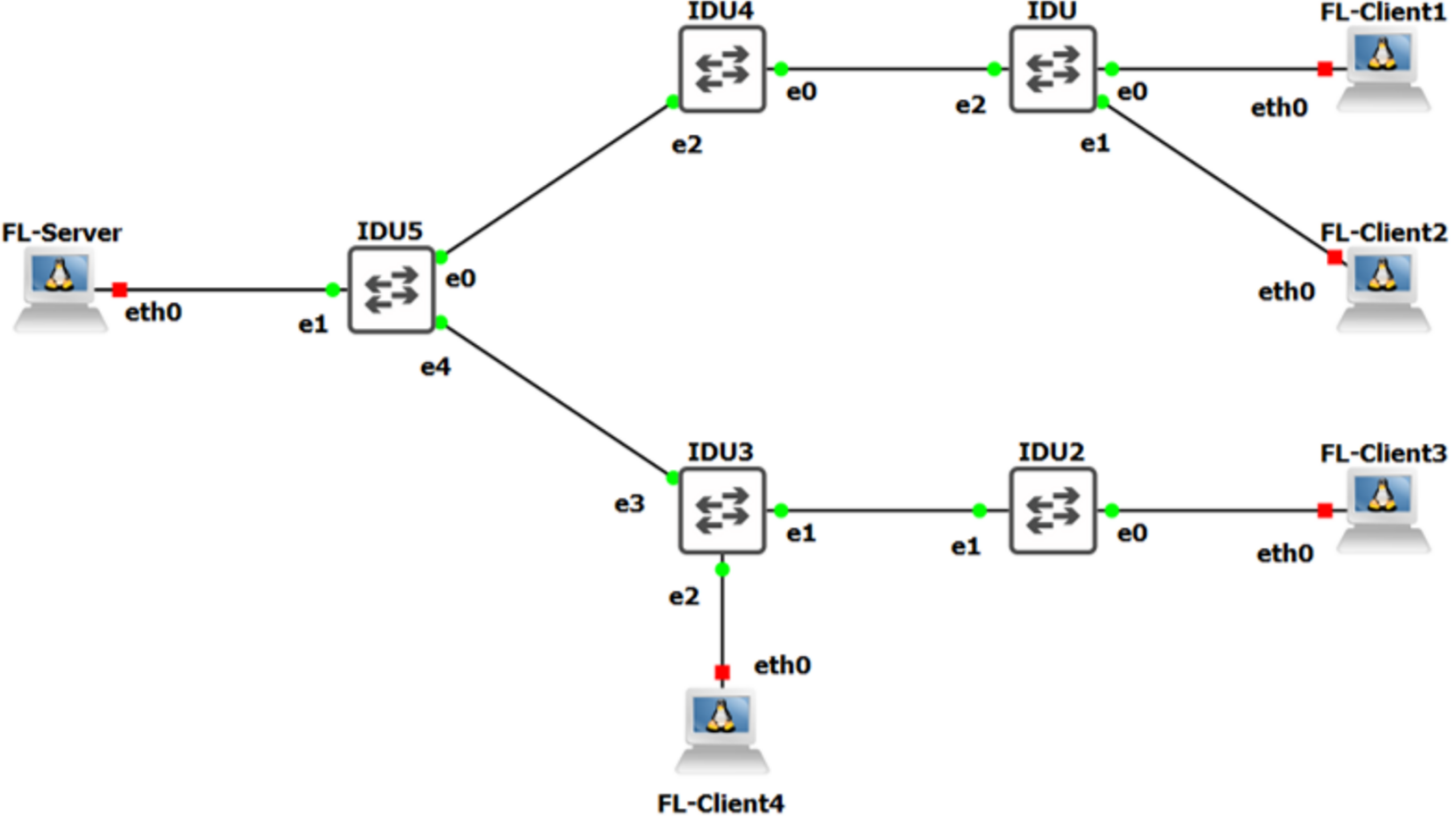
The replica of proposed framework implemented in virtual environment.

In this proposed approach, the regular backhaul traffic remains undisturbed, employing a straightforward VPN architecture. A dedicated VPN is established over the conventional traffic, creating a distinct tunnel for transmitting and receiving data within the federated network. The implementation of the FL framework for a wireless backhaul network, utilizing VPN, has been successfully accomplished through the GNS3 virtual environment emulator. This emulator simplifies the tasks of designing, constructing, emulating, configuring, testing, and resolving issues in both virtual and actual networks, eliminating the necessity for physical hardware. The network controller is constructed based on the OpenDaylight (ODL) architecture, utilizing Open vSwitches (OvS) and layer three switches/routers to replicate the proposed topology in the simulation environment. The experiments, illustrated with a set of four clients, involve each client contributing its own HFMD biosensor data. To implement the federated learning strategy, the Flower framework (Beutel et al., 2022) has been installed and properly configured on both the client and server sides, executing the FedAvg, Q-FedAvg, and FedProx algorithms. The training process on the clients involves utilizing the HFMD dataset with neural networks, as extensively explained in Mahmood et al. (2022). In Fig. 17 the simulation setup is explained.

#### Simulation settings

Table 5 provides a comprehensive overview of the diverse simulation settings that underwent testing and evaluation within the framework proposed in this study. These settings are carefully examined and then compared with the outcomes derived from the developed benchmark. The analysis involves a thorough exploration of the performance and efficiency of the proposed framework under different conditions, aiming to establish a robust basis for comparison with the established benchmark.

**Table 5:** Simulation settings.

**Setting No.**	**X-axis**	**Y-axis**	**Z-axis**
1	FedAvg	2.4 GHz, 8GB, No GPU	Terahertz
2	FedAvg	2.4 GHz, 8GB, No GPU	mmWave
3	FedAvg	2.4 GHz, 8GB, No GPU	Microwave
4	FedProx	2.4 GHz, 8GB, No GPU	Terahertz
5	FedProx	2.4 GHz, 8GB, No GPU	mmWave
6	FedProx	2.4 GHz, 8GB, No GPU	Microwave
7	Q-FedAvg	2.4 GHz, 8GB, No GPU	Terahertz
8	Q-FedAvg	2.4 GHz, 8GB, No GPU	mmWave
9	Q-FedAvg	2.4 GHz, 8GB, No GPU	Microwave

In standard practice, a single algorithm is chosen for FL model learning. we explored three distinct learning algorithms in conjunction with THz communication networks at mm-wave and microwave frequencies. The approach involves a three-dimensional grid search, where each dimension has three parameters. Local device parameters are fixed to CPU, GPU, and RAM. On the *x*-axis, we represented FL algorithm choices: FedAvg, Q-FedAvg, and FedProx. Wireless channel options include terahertz (10 Gbps), mm-wave (2 Gbps), and microwave (1 Gbps). Additionally, 20% of the total comprises THz (VPN) at 2000 Mbps, mmW at 400 Mbps, and MW at 200 Mbps.

The optimization results aim to improve the efficiency of both the algorithms and the network, ultimately identifying the best possible combination.

#### Federated aggregation learning algorithms

Various optimization strategies like federated averaging (FedAvg) and FedProx facilitate federated learning. FedProx, a modified version of FedAvg, considers statistical and system heterogeneity among end-devices. FedAvg uses stochastic gradient descent (SGD) on devices to get local model weights, which are then averaged at the edge computing server at the base station (BS).

FedProx follows a process similar to FedAvg but with a distinction in how the local device minimizes the objective function. It incorporates the FedAvg objective function and introduces an additional proximal term. This adjustment in FedProx aims to reduce the impact of non-independent and identically distributed (non-IID) device data on the global learning model. Unlike FedAvg, which lacks theoretical convergence guarantees, FedProx demonstrates theoretical convergence.

Unlike FedAvg, q-fair federated learning uses weighted averaging. In q-federated learning, local devices with high empirical loss are given a higher relative weight, introducing weighted averaging to enhance fairness and minimize training accuracy variance.

## Results and Discussion

In ‘Experiment Results’, we have explained the acheived results throught the centralized ML approach where the best accuracy is 80%. Now in this section we present the results acheived in federated network. and then we compare with FL results and draw the conclusion either the FL approach is giving the acceptable result or not. We presented the three best results, and our selection is focused on choosing the top-performing outcome among these three. The criteria for identifying the best results involve maintaining a constant *y*-axis. Specifically, from the *x*-axis, we opted for FedAvg as the learning algorithm. This algorithm is then tested across various *z*-axis options, encompassing terahertz, mmWave, and microwave, to comprehensively assess its performance under different conditions (see Table 6).

**Table 6:** Experiment no. 1 results with FedAvg Algo.

**Z-axis**	**Accuracy of Client 1**	**Accuracy of Client 2**	**Accuracy of Client 3**	**Accuracy of Client 4**	**Conv. time**
Microwave	81	57	70	76	51 seconds
mmWave	86	69	46	84	41 seconds
Terahertz	98	83	88	65	33 s

Likewise, in Test (No. 2) we selected FedProx as the learning algorithm. This algorithm was subsequently evaluated with different *z*-axis options, such as terahertz, mmWave, and microwave, to thoroughly assess its performance across various conditions (refer to Table 7).

**Table 7:** Experiment no. 2 results with FedProx Algo.

**Z-axis**	**Accuracy of Client 1**	**Accuracy of Client 2**	**Accuracy of Client 3**	**Accuracy of Client 4**	**Conv. Time**
Microwave	72	56	83	17	51 s
mmWave	84	90	67	52	39 seconds
Terahertz	72	70	86	71	42 seconds

In the most recent test, (No. 3) we chose Q-FedAvg as the learning algorithm. This algorithm was then evaluated with different *z*-axis options, such as terahertz, mmWave, and microwave, to thoroughly examine its performance under various conditions (refer to Table 8).

**Table 8:** Experiment no. 2 results with Q-FedAvg Algo.

**Z-axis**	**Accuracy of Client 1**	**Accuracy of Client 2**	**Accuracy of Client 3**	**Accuracy of Client 4**	**Conv. time**
Microwave	82	58	95	35	55 s
mmWave	85	70	81	34	38 s
Terahertz	94	73	23	97	37 Seconds

In this section, we presented the rankings based on our analysis, where we leveraged the insights gained in the previous section. Our investigation reveals that the highest accuracy is attained through the application of the FedAvg algorithm for parameter learning, utilizing the terahertz bandwidth. This results in a remarkably fast convergence of the model within a mere 33 s. The second-best performance is observed when employing the QFedAvg algorithm for learning, also utilizing the terahertz bandwidth. In this case, the model converges in 37 s, showcasing the efficacy of this approach. For a comprehensive overview of the rankings, please refer to Table 9, where we detailed the performance metrics of various algorithms in or study.

**Table 9:** Ranking of the experiments.

**Learning Algo**	**Channel BW**	**Average accuracy**	**Conv. time**	**Ranking**
FedAvg	Microwave	71	51 s	6
FedAvg	mmWave	71.25	41 s	5
Fed Avg	Terahertz	83.5	33 s	1
FedProx	Microwave	57	51 s	9
FedProx	mmWave	73.25	39 s	4
FedProx	Terahertz	74.75	42 s	3
Q-FedAvg	Microwave	67.5	55 s	8
Q-FedAvg	mmWave	67.5	38 s	7
Q-FedAvg	Terahertz	79.25	37 s	2

## Conclusion

Implementing federated learning across wireless networks requires the essential establishment of successful interaction between end-devices and the aggregation server. At the aggregation server, three distinct learning algorithms—namely, FedAvg, FedProx, and Q-FedAvg—have been utilized.

An additional influential factor impacting the efficacy of federated learning is system heterogeneity, as emphasized by Khan et al. (2021). End-devices characterized by system heterogeneity produce varied local learning models. The amalgamation of these models at the global server may result in weight divergences between the end-devices and the aggregation server.

Throughout our experiments, we maintained consistent device specifications. However, it is crucial to recognize that system heterogeneity can significantly influence the convergence performance of the global model. For example, if a device with lower specifications is assigned resource-intensive tasks, such as image processing, it can negatively impact the overall convergence time.

Devices with limited computational resources may encounter challenges in computing their local learning models within the specified deadline, particularly when the number of local iterations is high. Consequently, for devices with restricted computational resources, it becomes imperative to execute the local model for a reduced number of iterations. Nevertheless, it is essential to acknowledge that running a local model for fewer iterations generally results in diminished accuracy for the global federated learning model (McMahan et al., 2017).

The best accuracy is achieved using FedAvg and THz for the communication channel, with convergence times varying from 55 s to 24 s for FedAvg and transitioning from THz to downgraded MW. This underscores the critical importance of a higher bandwidth communication link. Determining device rules cannot heavily rely on the dataset alone. Security is enhanced through a three-step approach: a private network within the telecom network, a private network with licensed frequency channels, and a private network with a licensed band, further fortified by VPN-based security.

##  Supplemental Information

10.7717/peerj-cs.2422/supp-1 Supplemental Information 1 Data.

10.7717/peerj-cs.2422/supp-2 Supplemental Information 2 Code.

## Additional Information and Declarations

### Data Availability

The following information was supplied regarding data availability: The raw data and code are available in the Supplemental Files.
